# Patients’ Understanding of Health Information in Online Medical Records and Patient Portals: Analysis of the 2022 Health Information National Trends Survey

**DOI:** 10.2196/62696

**Published:** 2025-05-30

**Authors:** Katerina Andreadis, Nancy Buderer, Aisha Tene Langford

**Affiliations:** 1 Department of Population Health NYU Grossman School Of Medicine New York, NY United States; 2 Nancy Buderer Consulting LLC Oak Harbor, OH United States; 3 Department of Family Medicine and Public Health Sciences Wayne State University School of Medicine Detroit, MI United States

**Keywords:** patient portals, health literacy, mobile apps, digital health, online medical records, online health information, patient-focused care, health technology

## Abstract

**Background:**

The 21st Century Cures Act mandated instant digital access for patients to see their test results and clinical notes (eg, via patient portals). Entirely using and understanding such health information requires some degree of personal health literacy.

**Objective:**

This study aims to assess the associations between ease of understanding online health information and various factors, including sociodemographics, health-related variables, numeracy, and technology-related factors.

**Methods:**

This cross-sectional study used data from the National Cancer Institute’s 2022 Health Information National Trends Survey (HINTS), a nationally representative survey of US adults that tracks individuals’ access and use of their health information. Data was collected from March to December 2022. The survey was conducted across various US settings using a stratified multistage sampling technique to ensure national representation. Our analysis included 3016 respondents with data for all variables of interest. We conducted bivariate and multivariate analyses to assess the odds of finding health information in online medical records or patient portals as “very easy” to understand compared with “not very easy.”

**Results:**

In the multivariate analysis, age group (with the 35-49 years group being 1.9 times more likely compared to the ≥75 years group; *P*=.03), female birth sex (1.4 times more likely; *P*=.04), ease of understanding medical statistics (8.5 times more likely for those finding it “very easy”; *P*<.001), patient-provider communication score (increase of 1.1 odds per 1 unit increase; *P*<.001), and mode of accessing online records (1.8 times more likely via an app and 1.4 times more likely via both an app and website, *P*=.01 and *P*=.003, respectively, versus using a website alone) were significant predictors for finding health information “very easy” to understand.

**Conclusions:**

Sociodemographic factors, numeracy, patient-provider communication, and method of accessing online records were associated with ease of understanding health information in online medical records or patient portals. Findings from this study may inform interventions to make patient portals and online medical records more patient-centered and easier to navigate.

## Introduction

The landscape of health care has been transformed by the integration of digital tools, such as electronic health records, patient portals, and telemedicine platforms, driven by technological advancements, regulatory policies, and a shift toward more patient-focused care [1-4]. One such innovation is the patient portal, a secure online platform that allows 24-hour access to personal health information [5]. Patient portals have been associated with increased patient satisfaction, engagement, empowerment, and improved health outcomes [6]. There have been substantial efforts toward developing and integrating patient portals into health care systems, supported by policies such as the Health Information Technology for Economic and Clinical Health (HITECH) Act, which promoted the adoption of certified electronic health records with patient engagement capabilities, largely implemented via patient portals [7].

Despite these initiatives aimed at increasing patient access to their electronic health information, disparities in access and use persist. According to a recent analysis of Health Information National Trends Survey (HINTS) data, while the overall use of patient portals has steadily increased since 2014, only 60% of US residents were offered a patient portal by their health care provider, and just 40% had accessed one in 2020 [8,9]. Disparities are even more prominent among certain demographic groups, with Black and Hispanic individuals being less likely to be offered online access to their medical records by their health care provider compared to White patients [10]. Various research studies have documented disparities in patients who are male, Hispanic, hold less than a college degree, are Medicaid recipients, lack a regular provider, or do not have the internet to access a patient portal [11-13].

While efforts have been made to address these disparities in patient portal uptake [14], other initiatives, like the 21st Century Cures Act (Cures Act), which was signed into law in 2016, attempt to increase the accessibility, availability, and transparency of health data [4]. The Cures Act Final Rule (Cures Rule), enacted in 2020, mandates the immediate electronic availability of test results and clinical notes, with its information-blocking provisions taking effect in 2021 [15]. The Cures Act aims to empower patients in making health decisions by eliminating the “waiting period” for releasing health records [16-18]. While the Cures Act expands information availability, new potential challenges arise, particularly in the context of health literacy, at both personal and organizational levels. Healthy People 2030 defines personal health literacy as “the degree to which individuals have the ability to find, understand, and use information and services to inform health-related decisions and actions for themselves and others,” and organizational health literacy as “the degree to which organizations equitably enable individuals to find, understand, and use information and services to inform health-related decisions and actions for themselves and others” [19]. As patient portals and other health technologies are increasingly used, patients may face the growing expectation to independently interpret their health information, while organizations have an increasing responsibility to make health information easier to access and navigate. While increased accessibility to health information can be empowering, it is not without potential drawbacks. As shown with the adoption of patient portals, informatics interventions can unintentionally exacerbate disparities through inequitable access, barriers to usability, distrust in technology and the medical system, and lower adherence and effectiveness among vulnerable or historically underserved groups [3].

Recent studies have begun evaluating the implications of the Cures Act. A study using 2 years of data collected between 2020 and 2022 from Vanderbilt University Medical Center showed that 45.6% of patients reviewed their test results before their clinicians did, as compared to 11.5% of patients before the Cures Act mandate. An even more substantial rise was related to patients reviewing clinical results previously classified for release after 14 days, with patient review increasing from 4.7% to 56.6% [20]. Another study evaluating both portal access and use following the Cures Act reported that patients who identified as non-Hispanic Black, spoke Spanish, or had public insurance were less likely to read their clinical notes [21].

Despite the increasing availability of online health information, gaps remain in our understanding of how accessible and comprehensible this information truly is for patients. Greater accessibility does not inherently equate to ease of understanding—patients may have access to their medical records and test results, but without sufficient health literacy or support, they may struggle to interpret the information in a meaningful way. While many studies have examined portal use and disparities in access, fewer have specifically evaluated patients’ perceived ease of understanding their online health information. This study addresses this gap by examining the association between patients’ ease of understanding their online health information and various factors, including sociodemographics, health-related variables, numeracy, and technology-related variables. Through this analysis, we aim to offer insights that could guide the development of more inclusive and effective digital health strategies, contributing to the ongoing discourse on health equity and digital literacy [22-24].

## Methods

### Study Design and Data Source

This study used data from HINTS 6, collected in 2022, to explore bivariate and multivariate associations between the ease with which respondents understood health information in their online medical records or patient portals and various sociodemographic, health-related, and technology-related factors. HINTS collects data from a nationally representative sample of the US population, focusing on trends in health communication and health IT [25]. The HINTS 6 (2022) survey was administered via both mail and the web, using a two-stage sampling design [25,26].

### Participants

The HINTS 6 dataset originally included 6252 respondents. The inclusion criteria for this study required respondents to have an online medical record or patient portal offered by their health care provider or insurer. Respondents were excluded if they had missing data, indicated not having an online medical record or patient portal offered by a health care provider or insurer, or reported accessing their online medical record or patient portal zero times in the question “How many times did you access your online medical record or patient portal in the last 12 months?” We further eliminated respondents if they were missing data on the question “How easy or difficult was it to understand the health information in your online medical record or patient portal?” Finally, only respondents with data for all variables of interest were included in bivariate and multivariate analyses. After applying these criteria, the final sample size for analysis was 3016 respondents.

### Measures and Outcomes

The primary outcome variable was the ease of understanding health information in the online medical record or patient portal. Response options were categorized as “very easy” versus “not very easy” (the latter combining “somewhat easy,” “somewhat difficult,” and “very difficult” responses). Independent variables included a range of sociodemographic, health-related, and technology-related factors.

Sociodemographic variables included age, birth sex, education level, and race/ethnicity. Numeracy was evaluated through a self-assessed measure of ease in understanding medical statistics. Health-related factors encompassed general health status, self-assessed ability to manage health, and specific conditions, including deafness, diabetes, high blood pressure, heart conditions, lung disease, depression, and cancer history. The patient-provider communication score integrated 7 variables assessing the clarity of communication, the provider’s ability to listen, how frequently medical information was explained in an understandable way, involvement in decision-making, time spent during visits, ensuring the patient understood follow-up instructions, and support for managing health outside of clinical visits. Provider encouragement to use a patient portal was not included in this score. Because these 7 items were intended to measure an overall construct of communication quality, we did not separately test correlations among them. Technology factors considered how respondents accessed online records and used health and wellness apps and wearable devices for tracking health. Portal use was captured by activities such as viewing results, downloading information, sending information to third parties, and viewing clinical notes. Finally, patient portal features included the organizational source of portals, the use of multiple portals, and the use of portal organizer apps (detailed description of included measures in Multimedia Appendix 1).

### Statistical Analysis

We used weighted logistic regression with jackknife variance estimation to account for the complex multistage survey design recommended by HINTS (which provides an overall sample weight and 50 replicate weights) [26]. We first ran bivariate logistic regression models to examine each independent variable’s association with the outcome. Variables meeting a prespecified *P*<.10 criterion were then considered for multivariable logistic regression, where backward elimination was used to retain only variables with a *P* value <.05. Parameter estimation was carried out using iterative maximum likelihood methods (Newton-Raphson algorithm). We selected reference groups (eg, younger adults, male) consistent with common epidemiological conventions. While an alternative can highlight disadvantages differently, we opted for a familiar baseline that mirrors previous health disparities research. Results are presented as the odds ratio (OR; 95% CI) for the odds of “very easy” versus “not very easy” (“not very easy” is a combination of “somewhat easy,” “somewhat difficult,” and “very difficult”) and *P* values. CIs not encompassing the value 1 were considered significant. All analyses were performed using SAS software, version 9.4, SAS/STAT 14.1 (SAS Institute).

### Ethical Considerations

The HINTS 6 general population survey was designated “exempt research” under 45 CFR 46.104 and approved by the Westat Institutional Review Board on May 10, 2021 (project 6632.03.51), with a subsequent amendment approved on November 24, 2021 (amendment 3597). HINTS 6 also received a “Not Human Subjects Research” determination from the National Institutes of Health (NIH) Office of Institutional Review Board Operations on August 16, 2021 (iRIS reference 562715) [27]. A written invitation letter describing risks and benefits was included with each instrument, and participants were informed of their right to opt out of or skip any or all items on the survey. Further documentation of written consent was waived due to the survey being designated as low risk. Both modes of the survey (paper and online) were offered in English or Spanish. All groups received a US $2 prepaid monetary incentive to encourage participation. Respondents in the control group were offered a bonus incentive to complete the survey online. Further details can be seen in the HINTS 6 methodology report [28].

## Results

A total of 3016 respondents met the inclusion criteria and had complete data for this analysis. The distribution of responses to the question, “How many times did you access your online medical record or patient portal in the last 12 months?” was as follows: 917 (weighted 30.9%) respondents accessed their records 1-2 times, 965 (weighted 32.1%) respondents accessed 3-5 times, 496 (weighted 16.4%) respondents accessed 6-9 times, and 638 (weighted 20.6%) respondents accessed 10 or more times. Of the 3016 respondents, 46.7% (weighted) indicated that it was “very easy” to understand the health information in their online medical record or patient portal, while 53.3% (weighted) reported finding it “not very easy.”

Our bivariate analyses revealed a range of factors associated with patients’ ease of understanding their online health information (Table 1).

**Table 1:** Bivariate associations with ease of understanding online medical records or patient portals.

			How easy or difficult was it to understand the health information in your online medical record or patient portal?							
			Very easy (n=1403)^a^, weighted % of row (SE)		Not very easy^b^ (n=1613)^c^, weighted % of row (SE)		Odds ratio for very easy (95% CI)		*P* value	
**Age group (years)**										.04
	18-34	44.6 (3.3)		55.4 (3.3)		Reference		—^d^		
	35-49	52.0 (2.9)		48.0 (2.9)		1.3 (1.0-1.8)		.07		
	50-64	45.5 (2.8)		54.5 (2.8)		1.0 (0.7-1.5)		.84		
	65-74	45.3 (3.0)		54.7 (3.0)		1.0 (0.7-1.5)		.89		
	≥75	39.3 (3.6)		60.7 (3.6)		0.8 (0.5-1.2)		.30		
**Birth sex**										
	Female	49.6 (2.2)		50.4 (2.2)		1.3 (1.0-1.8)		.08		
	Male	42.8 (2.7)		57.2 (2.7)		Reference		—		
**Race/ethnicity**										.19
	Non-Hispanic White	46.3 (1.9)		53.7 (1.9)		Reference		—		
	Non-Hispanic Black or African American	54.3 (3.8)		45.7 (3.8)		1.4 (1.0-1.9)		.06		
	Hispanic	48.6 (4.6)		51.4 (4.6)		1.1 (0.8-1.6)		.62		
	Non-Hispanic Asian	41.7 (9.3)		58.3 (9.3)		0.8 (0.4-1.8)		.63		
	Non-Hispanic Other	37.8 (7.6)		62.2 (7.6)		0.7 (0.4-1.4)		.31		
**Education**										
	College graduate	48.2 (1.9)		51.8 (1.9)		1.1 (0.9-1.4)		.32		
	Not college graduate	45.5 (2.2)		54.5 (2.2)		Reference		—		
**Ease of understanding medical statistics**										<.001
	Very easy	78.1 (2.6)		21.9 (2.6)		10.2 (6.8-15.2)		<.001		
	Easy	44.2 (1.8)		55.8 (1.8)		2.3 (1.5-3.3)		<.001		
	Hard	25.9 (3.4)		74.1 (3.4)		Reference		—		
**General health**										.02
	Excellent or very good	50.6 (2.2)		49.4 (2.2)		1.3 (1.0-1.8)		.09		
	Good	42.3 (2.0)		57.7 (2.0)		0.9 (0.7-1.3)		.72		
	Fair or poor	43.7 (3.5)		56.3 (3.5)		Reference		—		
**Confidence in own ability to take care of health**										.001
	Completely or very	50.5 (1.8)		49.5 (1.8)		1.8 (0.6-5.9)		.33		
	Somewhat or little	36.6 (3.1)		63.4 (3.1)		1.0 (0.3-3.5)		.98		
	Not at all	36.2 (13.4)		63.8 (13.4)		Reference		—		
**Deaf**										
	Yes	36.8 (4.7)		63.2 (4.7)		0.6 (0.4-1.0)		.04		
	No	47.3 (1.6)		52.7 (1.6)		Reference		—		
**Diabetes**										
	Yes	44.6 (3.3)		55.4 (3.3)		0.9 (0.6-1.3)		.56		
	No	47.1 (1.9)		52.9 (1.9)		Reference		—		
**High blood pressure**										
	Yes	46.3 (2.4)		53.7 (2.4)		1.0 (0.8-1.3)		.86		
	No	46.9 (2.0)		53.1 (2.0)		Reference		—		
**Heart condition**										
	Yes	40.7 (4.5)		59.3 (4.5)		0.8 (0.5-1.1)		.17		
	No	47.1 (1.6)		52.9 (1.6)		Reference		—		
**Lung disease**										
	Yes	43.2 (4.5)		56.8 (4.5)		0.9 (0.6-1.2)		.40		
	No	47.2 (1.6)		52.8 (1.6)		Reference		—		
**Depression**										
	Yes	49.1 (2.9)		50.9 (2.9)		1.2 (0.9-1.5)		.29		
	No	45.6 (1.8)		54.4 (1.8)		Reference		—		
**Ever had cancer**										
	Yes	43.7 (3.2)		56.3 (3.2)		0.9 (0.7-1.1)		.30		
	No	47.0 (1.6)		53.0 (1.6)		Reference		—		
**Patient-provider communication score, median (IQR)**			25.6 (21.6-27.5)		22.3 (18.8-26.2)		1.14 (1.10-1.18)^e^		<.001	
**How online records accessed**										.02
	App	53.8 (4.1)		46.2 (4.1)		1.6 (1.1-2.4)		.02		
	Website	41.8 (2.3)		58.2 (2.3)		Reference		—		
	Both app and website	49.9 (2.6)		50.1 (2.6)		1.4 (1.1-1.8)		.02		
**Records online: view results**										
	Yes	47.8 (1.6)		52.2 (1.6)		1.8 (1.1-2.7)		.01		
	No	34.1 (4.8)		65.9 (4.8)		Reference		—		
**Records online: download health information**										
	Yes	47.9 (2.6)		52.1 (2.6)		1.1 (0.8-1.4)		.59		
	No	46.1 (2.0)		53.9 (2.0)		Reference		—		
**Records online: send to third party**										
	Yes	52.4 (3.2)		47.6 (3.2)		1.3 (1.0-1.8)		.05		
	No	45.2 (1.8)		54.8 (1.8)		Reference		—		
**Records online: view notes**										
	Yes	50.0 (1.9)		50.0 (1.9)		1.6 (1.2-2.2)		.004		
	No	38.5 (3.0)		61.5 (3.0)		Reference		—		

^a^Weighted percent of all respondents: 46.7%.

^b^Not very easy is a combination of somewhat easy, somewhat difficult, and very difficult.

^c^Weighted percent of all respondents: 53.3%.

^d^Not applicable.

^e^Increase in odds per 1 unit increase in score.

Individuals who reported “very easy” understanding of medical statistics (78.1% vs 25.9%) had higher odds of finding their health information “very easy” to understand (OR 10.2, 95% CI 6.8-15.2; *P*<.001). Deaf individuals, however, were less likely to find it “very easy” (OR 0.6, 95% CI 0.4-1.0; *P*=.04) compared to their nondeaf counterparts (36.8% vs 47.3%). A 1-point increase in the patient-provider communication score was associated with increased odds of finding health information “very easy” (OR 1.14, 95% CI 1.10-1.18; *P*<.001). In addition, accessing online records via an app (OR 1.6, 95% CI 1.1-2.4; *P*=.02) or having the ability to view test results (OR 1.8, 95% CI 1.1-2.7; *P*=.01) and clinical notes (OR 1.6, 95% CI 1.2-2.2; *P*=.004) each increased the odds of reporting information as “very easy” to understand.

The multivariate analysis revealed further relationships (Table 2). Those in the 35-49 years age group were 1.9 (95% CI 1.25-2.80; *P*=.03) times more likely to find health information “very easy” compared to the ≥75 years age group. Female respondents were 1.4 (95% CI 1.02-2.03; *P*=.04) times more likely to find health information “very easy” to understand than male respondents. Additionally, higher patient-provider communication scores (OR 1.1, 95% CI 1.09-1.16; *P*<.001) and ease of understanding medical statistics (OR 8.5, 95% CI 5.60-12.78; *P*<.001) were significantly associated with ease of understanding health information. Modes of accessing online records also remained significant: respondents accessing via an app were 1.8 (95% CI 1.19-2.68; *P*=.01) times more likely to find it “very easy” compared to those using a website alone, and those accessing through both an app and a website were 1.4 (95% CI 1.03-1.94; *P*=.003) times more likely to find it “very easy.”

**Table 2:** Multivariate associations with ease of understanding online medical records or patient portals^a^.

			“Very easy” vs “Not very easy,” adjusted odds ratio (95% CI)		*P* value
**Age group (years)**					.05
	18-34	1.5 (0.93-2.51)		.09	
	35-49	1.9 (1.25-2.80)		.003	
	50-64	1.4 (0.94-2.03)		.09	
	65-74	1.4 (0.89-2.23)		.14	
	≥75	Reference		—^b^	
**Birth sex**					
	Female	1.4 (1.02-2.03)		.04	
	Male	Reference		—	
Patient-provider communication score			1.1 (1.09-1.16)^c^		<.001
**Ease of understanding medical statistics**					<.001
	Very easy	8.5 (5.60-12.78)		<.001	
	Easy	2.0 (1.31-2.94)		.002	
	Hard	Reference		—	
**How online records were accessed**					.01
	App	1.8 (1.19-2.68)		.01	
	Website	Reference		—	
	Both app and website	1.4 (1.03-1.94)		.03	

^a^Factors that were bivariately significant with *P*<.10 were attempted in the model. Using backward elimination, factors were removed one by one until all the remaining factors in the model were significant with *P*<.05.

^b^Not applicable.

^c^Increase in odds per 1 unit increase in score.

## Discussion

### Principal Results

The goal of this study was to understand the factors associated with ease of understanding health information in the patient portal or online medical record. In summary, ease of understanding medical statistics, patient-provider communication scores, female birth sex, age, and those accessing online records through an app or both an app and a website were significantly more likely to find health information in online medical records “very easy” to understand.

### Comparison With Prior Work

Prior studies have evaluated age and sex differences with regard to initial uptake and continued use of patient portals and online medical records [29,30]. In our study, older adults, especially those 75 years or older, found it more challenging to understand health information in their patient portal or online medical record, compared to those aged 35-49 years. This finding might be due to differences in comfort with technology/eHealth or the limited support provided to patients on using these platforms. Emerging interventions to support older patients with technology include the use of digital health navigators and general patient navigators [14,31,32]. Birth sex differences were also evident, with our findings showing that women found it easier to understand health information compared to men, which could be reflective of findings from studies showing that women are more likely than men to use patient portals [33] or be more active telemedicine users [34]. With regard to the other sociodemographic factors we evaluated, including race/ethnicity and educational attainment, these were not associated with ease of understanding. While numerous studies have examined various aspects of health information comprehension, such as test result interpretation, few have specifically evaluated the *perceived ease* of understanding health information within patient portals and online medical records. These are important distinctions (ie, initial uptake, continued use, and actual ease of understanding); thus, our findings contribute new knowledge to this literature.

Perceived patient-provider communication was another significant factor, suggesting that better communication correlates with an increased ease of understanding health information. This is in line with studies showing that patients with higher perceptions of provider communication had an increased likelihood of both being offered and using a portal account [11]. Similarly, Sisk et al’s [35] evaluation of patient portal disparities using HINTS data reported the pivotal role of clinician encouragement in facilitating access to the electronic medical record via online patient portals. Additionally, research on patient engagement with electronic health records and patient portals has shown that a lack of provider encouragement is a significant barrier to portal use, whereas strong patient-provider communication serves as a key facilitator [36]. Our study contributes new insights into this dialogue, revealing that participants with a higher perceived quality of patient-provider communication reported greater ease in understanding health information. Our findings underscore the vital role of health care providers in enhancing patient understanding and engagement with health information via patient portals.

Furthermore, our study established a strong association between the ease of understanding medical statistics and the ease of understanding health information in electronic medical records or patient portals. This finding might be explained by the fact that test results (eg, for cholesterol, hemoglobin A_1c_) and risk probabilities are often presented via online medical records and patient portals. Our analysis shows that individuals who reported finding it “very easy” to understand medical statistics were 8.46 times more likely to effectively comprehend their health information in their patient portals or online medical records, which underscores the importance of numeracy and health literacy, and suggests that proficiency in both areas helps navigate and make sense of medical data in digital platforms. Echoing Zikmund-Fisher et al’s [37] research, our study confirms that health literacy and numeracy are necessary skills for patients aiming to understand their health information, such as when identifying abnormal test results. A different study by Di Tosto et al [38] suggested that portal use and engagement are not necessarily hindered by low levels of health literacy and numeracy, and instead, the focus should be on finding strategies to increase technology acceptance. Future work should explore personalized educational efforts and user-friendly design to bridge the gap in digital health literacy, empowering patients with the confidence needed to manage their health effectively. By integrating such strategies, health systems can significantly contribute to the overall effectiveness of patient portals as a tool for patient education and engagement.

In line with inclusive design principles, our study sheds light on the significant impact of the medium (eg, app, website) through which health information is accessed. Specifically, we found that patients who accessed their health information through a mobile app, or both an app and a website, compared to those who used a website alone, reported a notably higher ease of understanding, which is also a novel finding. This difference may be attributed to the more user-friendly and simplified interface of mobile apps, which are often designed with a focus on accessibility and ease of navigation. This finding highlights the importance of considering the user experience in the design of digital health tools. By prioritizing interfaces that cater to diverse user needs and preferences, digital health platforms can enhance patient engagement and comprehension. Ensuring that patients can easily access and understand their health information is crucial for empowering them to make informed decisions about their health care. This approach aligns closely with the principles of organizational health literacy, advocating for equitable access to health information and services.

A systematic review from 2020 noted that while patients prefer unrestricted access to their medical records, unrestricted access alone without further explanations may unintentionally offload responsibilities such as checking for medical errors onto patients [39]. Another study examining the effect of the Cures Act, enacted in 2021, reported that releasing results with immediate notification was followed by sharp increases in patient-initiated messaging, which could be indicative of increased patient responsibility [20]. Consistent with these findings, a large survey study found that 95.7% of patients preferred to receive test results immediately through the portal, even for abnormal results [40]. While this strong preference suggests that patients want direct access to their health information, many still take additional steps to interpret it, with 40% seeking further information after reviewing their results, often turning to internet searches [40]. This highlights the significant effort patients invest in understanding their health information independently. While the implementation of the Cures Act marked a significant step toward enhancing patient access to their medical records, greater accessibility does not inherently equate to ease of understanding health information in the online medical records and patient portals. One way to improve patients’ understanding of their health information in their patient portals and online medical records is through organizational health literacy strategies, such as the use of plain language and simplified visual tools. Additionally, it can be helpful for health systems to incorporate patient feedback as patient portals are being adapted over time. Building on prior studies, this work extends current knowledge by leveraging recent, nationally representative data from HINTS in the post–Cures Act era, examining patient-provider communication and mobile app use as key factors that may shape patients’ ease of understanding portal-based health information.

### Strengths, Limitations, and Future Directions

Strengths of the study include the use of a nationally representative survey designed to monitor health communication and IT trends among US adults and a robust set of health- and technology-related variables. However, some limitations must be noted. First, the data did not specify which sections of the patient portals or online medical records were considered when respondents assessed the ease of understanding. For example, test results, upcoming appointments, messaging providers, or viewing after-visit summaries are common sections of a patient portal account [5]. Similarly, we are not sure of the context in which patients were thinking with regard to understanding medical statistics, which could include genetic risk scores or probabilities of side effects. We also do not have information on the patients’ English fluency or any measure of cognition, both of which could affect the patients’ ability to interpret and use information from patient portals. Future research should further explore what patients consider facilitators and barriers to their ease of understanding of health information in their online medical records and patient portals, as well as examine whether ease of understanding changes with frequency of access.

### Conclusions

Despite the universal availability of health data, our findings suggest that the availability of online health data alone does not ensure understanding, particularly for older patients, male patients, those with lower numeracy skills, those with lower perceived patient-provider communication, and those accessing their online medical record through a website only. Findings from this study may inform interventions to make patient portals and online medical records more patient centered and easier to navigate.

## Appendix

Multimedia Appendix 1 Detailed description of study variables.

## Abbreviations

HINTS: Health Information National Trends Survey
HITECH: Health Information Technology for Economic and Clinical Health
NIH: National Institutes of Health
OR: odds ratio
