# Supplementary material for: Patients’ Understanding of Health Information in Online Medical Records and Patient Portals: Analysis of the 2022 Health Information National Trends Survey
Source: J Med Internet Res. 2025 May 30;27:e62696. doi: 10.2196/62696 (PMC12166317; doi:10.2196/62696)
Supplement: Multimedia Appendix 1 [file jmir_v27i1e62696_app1.docx]

**Appendix A: Detailed Description of Study Variables**

This Appendix provides a complete overview of all variables used in the analyses, including exact question wording, coding schemes, and how variables were collapsed or combined. All items below are taken from HINTS 6 (2022).

**A. Outcome Variable**

**Ease of Understanding Health Information**

- **Original Survey Question**: “How easy or difficult was it to understand the health information in your online medical record or patient portal?”
- **Response Options**: Very easy, Somewhat easy, Somewhat difficult, Very difficult
- **Coding for Analysis**:
  - **Very Easy** vs. **Not Very Easy** (collapsed “Somewhat easy,” “Somewhat difficult,” and “Very difficult”).

**B. Independent Variables included in Bivariate and/or Multivariate Analyses**

**1. Age Group**

- **Original Variable**: Continuous age in years.
- **Coding for Analysis**: Categorized into:
  - 18–34, 35–49, 50–64, 65–74, 75+

**2. Birth Gender**

- **Original Survey Question**: “On your original birth certificate, were you listed as male or female?”
- **Response Options**: Male, Female
- **Coding for Analysis**:
  - Male = reference
  - Female = comparison category

**3. Race/Ethnicity**

- **Original Survey Question**: What is your race? One or more categories may be selected.
- **Coding for Analysis**:
  - Non-Hispanic White (reference)
  - Non-Hispanic Black or African American
  - Hispanic
  - Non-Hispanic Asian
  - Non-Hispanic Other (includes American Indian/Alaska Native, multiracial, and others)

**4. Education**

- **Original Survey Question**: “What is the highest grade or level of schooling you completed?
- **Coding for Analysis**:
  - Not college graduate (reference)
  - College graduate (All other categories grouped)

**5. Ease of Understanding Medical Statistics (Self-Assessed Numeracy)**

- **Original Survey Question**: “In general, how easy or hard do you find it to understand medical statistics?”
- **Response Options**: Very easy, Somewhat easy, Somewhat difficult, Very difficult
- **Coding for Analysis**:
  - **‘Very easy,’ ‘Easy,’ ‘Hard’** (for bivariate and multivariable analyses, “Easy” included ‘somewhat easy,’ and “Hard” included ‘somewhat difficult’ or ‘very difficult,’ or as specified in the final model).

**6. General Health**

- **Original Survey Question**: “In general, would you say your health is…?”
- **Response Options**: Excellent, Very Good, Good, Fair, Poor
- **Coding for Analysis**: Combined into 3 categories:
  - (1) Excellent/Very Good,
  - (2) Good,
  - (3) Fair/Poor (reference)

**7. Confidence in Ability to Take Care of Own Health**

- **Original Survey Question**: “Overall, how confident are you in your ability to take care of your health?”
- **Response Options**: Completely confident, Very confident, Somewhat confident, A little confident, Not confident at all
- **Coding for Analysis**: Categorized into:
  - Completely/Very,’
  - ‘Somewhat/A Little,’
  - ‘Not at all’ (reference)

**8. Deaf**

- **Original Survey Question**: “Are you deaf or do you have serious difficulty hearing?”
- **Coding for Analysis**:
  - Yes
  - No (reference)

**9. Diabetes**

- **Original Survey Question**: “Has a doctor or other health professional ever told you that you have diabetes or high blood sugar?”
- **Coding**:
  - Yes
  - No (reference)

**10. High Blood Pressure**

- **Original Survey Question**: “Has a doctor or other health professional ever told you that you have high blood pressure or hypertension?”
- **Coding**:
  - Yes
  - No (reference)

**11. Heart Condition**

- **Original Survey Question**: “Has a doctor or other health professional ever told you that you have a heart condition such as heart attack, angina, or congestive heart failure?”
- **Coding**:
  - Yes
  - No (reference)

**12. Lung Disease**

- **Original Survey Question**: “Has a doctor or other health professional ever told you that you have chronic lung disease, asthma, emphysema, or chronic bronchitis?”
- **Coding**:
  - Yes
  - No (reference)

**13. Depression**

- **Original Survey Question**: “Has a doctor or other health professional ever told you that you have depression or anxiety disorder?”
- **Coding**:
  - Yes
  - No (reference)

**14. Ever Had Cancer**

- **Original Survey Question**: “Have you ever been diagnosed as having cancer?”
- **Coding**:
  - Yes
  - No (reference)

**15. Patient-Provider Communication Score**

- **Items Included (7 total)**:
  1. Chance to ask questions *(ChanceAskQuestions)*
  2. Attention to feelings/emotions *(FeelingsAddressed)*
  3. Involvement in decisions *(InvolvedDecisions)*
  4. Ensuring the patient understood next steps *(UnderstoodNextSteps)*
  5. Explaining in a clear way *(ExplainedClearly)*
  6. Spending enough time *(SpentEnoughTime)*
  7. Helping manage uncertainty *(DealWithUncertainty)*
- Response options included always, usually, sometimes, and never.
- Reponses were summed to created a total communication score, which was treated as a continuous variable; the final score ranges from X to Y.

**16. How Access Online Records**

- **Original Survey Question**: “In the past 12 months, did you access your online medical record or patient portal using…”
  - Mobile app on a smartphone or tablet
  - Website on a computer or laptop
  - Both app and website
- **Coding for Analysis**:
  - App only
  - Website only (reference)
  - Both app and website

**17. Records Online – View Results**

- **Original Survey Question**: “In the past 12 months, have you used your online medical record or patient portal to look up test results?”
- **Coding for Analysis**:
  - Yes
  - No (reference)

**18. Records Online – Download Health Information**

- **Original Survey Question**: “In the past 12 months, have you used your online medical record or patient portal to download your health information to your computer or mobile device, such as cell phone or tablet?
- **Coding for Analysis**:
  - Yes
  - No (reference)

**19. Records Online – Send Information to Third Party**

- **Original Survey Question**: “In the past 12 months, have you used your online medical record or patient portal to electronically send your medical information to a third party (such as another health are provider, a family member, or a smartphone health app)?
- **Coding for Analysis**:
  - Yes
  - No (reference)

**20. Records Online – View Notes**

- **Original Survey Question**: “In the past 12 months, have you used your online medical record or patient portal to view clinical notes (a health care provider’s written notes that describe your visit)?
- **Coding for Analysis**:
  - Yes
  - No (reference)
